# Supplementary material for: PEtab—Interoperable specification of parameter estimation problems in systems biology
Source: PLoS Comput Biol. 2021 Jan 26;17(1):e1008646. doi: 10.1371/journal.pcbi.1008646 (PMC7864467; doi:10.1371/journal.pcbi.1008646)
Supplement: S1 File — Detailed format description of PEtab version 1. (PDF) [file pcbi.1008646.s001.pdf]

PEtab data format specification

Format version: 1

This document explains the PEtab data format.

Purpose

Providing a standardized way for specifying parameter estimation problems in systems biology, especially for the case of Ordinary Differential Equation (ODE) models.

Overview

The PEtab data format specifies a parameter estimation problem using a number of text-based files (Systems Biology Markup Language (SBML) and Tab-Separated Values (TSV)), i.e.

- An SBML model [SBML]
- A measurement file to fit the model to [TSV]
- A condition file specifying model inputs and condition-specific parameters [TSV]
- An observable file specifying the observation model [TSV]
- A parameter file specifying optimization parameters and related information [TSV]
- (optional) A simulation file, which has the same format as the measurement file, but contains model simulations [TSV]
- (optional) A visualization file, which contains specifications how the data and/or simulations should be plotted by the visualization routines [TSV]

The following sections will describe the minimum requirements of those components in the core standard, which should provide all information for defining the parameter estimation problem.

Extensions of this format (e.g. additional columns in the measurement table) are possible and intended. However, while those columns may provide extra information for example for plotting, downstream analysis, or for more efficient parameter estimation, they should not affect the optimization problem as such.

General remarks - All model entities, column names and row names are case-sensitive - All identifiers must consist only of upper and lower case letters, digits and underscores, and must not start with a digit. - Fields in "[]" are optional and may be left empty.

SBML model definition

The model must be specified as valid SBML. There are no further restrictions.

Condition table

The condition table specifies parameters, or initial values of species and compartments for specific simulation conditions (generally corresponding to different experimental conditions).

This is specified as a tab-separated value file in the following way:

conditionId	[conditionName]	parameterOrSpeciesOrCompartmentId1	...	parameterOrSpeciesOrCompartmentId${n}
STRING	[STRING]	NUMERIC|STRING	...	NUMERIC|STRING
e.g.				
conditionId1	[conditionName1]	0.42	...	parameterId
conditionId2	...	...	...	...
...	...	...	...	...| ...

Row- and column-ordering are arbitrary, although specifying `conditionId` first may improve human readability.

Additional columns are *not* allowed.

Detailed field description

- `conditionId` [STRING, NOT NULL]

  Unique identifier for the simulation/experimental condition, to be referenced by the measurement table described below.

- `conditionName` [STRING, OPTIONAL]

  Condition names are arbitrary strings to describe the given condition. They may be used for reporting or visualization.

- `${parameterOrSpeciesOrCompartmentId1}`

  Further columns may be global parameter IDs, IDs of species or compartments as defined in the SBML model. Only one column is allowed per ID. Values for these condition parameters may be provided either as numeric values, or as IDs defined in the SBML model, the parameter table or both.

  - `${parameterId}`

  The values will override any parameter values specified in the model.

  - `${speciesId}`

  If a species ID is provided, it is interpreted as the initial concentration/amount of that species and will override the initial concentration/amount given in the SBML model or given by a preequilibration condition. If `NaN` is provided for a condition, the result of the preequilibration (or initial concentration/amount from the SBML model, if no preequilibration is defined) is used.

  - `${compartmentId}`

  If a compartment ID is provided, it is interpreted as the initial compartment size.

Measurement table

A tab-separated values files containing all measurements to be used for model training or validation.

Expected to have the following named columns in any (but preferably this) order:

observableId	[preequilibrationConditionId]	simulationConditionId	measurement	time
observableId	[conditionId]	conditionId	NUMERIC	NUMERIC|inf

observableId	[preequilibrationConditionId]	simulationConditionId	measurement	time
...	...	...	...	...

(wrapped for readability)

...	[observableParameters]	[noiseParameters]
...	[parameterId|NUMERIC[;parameterId|NUMERIC][...]]	[parameterId|NUMERIC[;parameterId|NUMERIC][...]]
...	...	...

Additional (non-standard) columns may be added. If the additional plotting functionality of PEtab should be used, such columns could be

...	[datasetId]	[replicateId]
...	[datasetId]	[replicateId]
...	...	...

where `datasetId` is a necessary column to use particular plotting functionality, and `replicateId` is optional, which can be used to group replicates and plot error bars.

Detailed field description

- `observableId` [STRING, NOT NULL, REFERENCES(observables.observableID)]

  Observable ID as defined in the observables table described below.

- `preequilibrationConditionId` [STRING OR NULL, REFERENCES(conditionsTable.conditionID), OPTIONAL]

  The `conditionId` to be used for preequilibration. E.g. for drug treatments, the model would be preequilibrated with the no-drug condition. Empty for no preequilibration.

- `simulationConditionId` [STRING, NOT NULL, REFERENCES(conditionsTable.conditionID)]

  `conditionId` as provided in the condition table, specifying the condition-specific parameters used for simulation.

- `measurement` [NUMERIC, NOT NULL]

  The measured value in the same units/scale as the model output.

- `time` [NUMERIC OR STRING, NOT NULL]

  Time point of the measurement in the time unit specified in the SBML model, numeric value or `inf` (lower-case) for steady-state measurements.

- `observableParameters` [NUMERIC, STRING OR NULL, OPTIONAL]

  This field allows overriding or introducing condition-specific versions of output parameters defined in the observation model. The model can define observables (see below) containing place-holder parameters which can be replaced by condition-specific dynamic or constant parameters. Placeholder parameters must be named `observableParameter${n}_${observableId}` with `n` ranging from 1 (not 0) to the number of placeholders for the given observable, without gaps. If the observable specified under `observableId` contains no placeholders, this field must be empty. If it contains `n > 0` placeholders, this field must hold `n` semicolon-separated numeric values or parameter names. No trailing semicolon must be added.

  Different lines for the same `observableId` may specify different parameters. This may be used to account for condition-specific or batch-specific parameters. This will translate into an extended optimization parameter vector.

  All placeholders defined in the observation model must be overwritten here. If there are no placeholders used, this column may be omitted.

- `noiseParameters` [NUMERIC, STRING OR NULL, OPTIONAL]

  The measurement standard deviation or `NaN` if the corresponding sigma is a model parameter.

  Numeric values or parameter names are allowed. Same rules apply as for `observableParameters` in the previous point.

- `datasetId` [STRING, OPTIONAL]

  The datasetId is used to group certain measurements to datasets. This is typically the case for data points which belong to the same observable, the same simulation and preequilibration condition, the same noise model, the same observable transformation and the same observable parameters. This grouping makes it possible to use the plotting routines which are provided in the PEtab repository.

- `replicateId` [STRING, OPTIONAL]

  The replicateId can be used to discern replicates with the same `datasetId`, which is helpful for plotting e.g. error bars.

Observables table

Parameter estimation requires linking experimental observations to the model of interest. Therefore, one needs to define observables (model outputs) and respective noise models, which represent the measurement process. Since parameter estimation is beyond the scope of SBML, there exists no standard way to specify observables (model outputs) and respective noise models. Therefore, in PEtab observables are specified in a separate table as described in the following. This allows for a clear separation of the observation model and the underlying dynamic model, which allows, in most cases, to reuse any existing SBML model without modifications.

The observable table has the following columns:

observableId	[observableName]	observableFormula	[observableTransformation]	noiseFormula	[noiseDistribution]
STRING	[STRING]	STRING	[lin(default)|log|log10]	STRING|NUMBER	[laplace|normal]
e.g.					
relativeTotalProtein1	Relative abundance of Protein1	observableParameter1_relativeTotalProtein1 * (protein1 + phospho_protein1)	lin	noiseParameter1_relativeTotalProtein1	normal
...	...	...	...	...	

Detailed field description:

- `observableId` [STRING]

  Any identifier which would be a valid identifier in SBML. This is referenced by the `observableId` column in the measurement table. Must be different from any existing model entity or parameter introduced elsewhere.

- [`observableName`] [STRING, OPTIONAL]

  Name of the observable. Only used for output, not for identification.

- `observableFormula` [STRING]

  Observation function as plain text formula expression. May contain any symbol defined in the SBML model or parameter table. In the simplest case just an SBML species ID or an `AssignmentRule` target.

  May introduce new parameters of the form `observableParameter${n}_${observableId}`, which are overridden by `observableParameters` in the measurement table (see description there).

- `observableTransformation` [STRING, OPTIONAL]

  Transformation of the observable and measurement for computing the objective function. Must be one of `lin`, `log` or `log10`. Defaults to `lin`. The measurements and model outputs are both assumed to be provided in linear space.

- `noiseFormula` [NUMERIC|STRING]

  Measurement noise can be specified as a numerical value which will default to a Gaussian noise model if not specified differently in `noiseDistribution` with standard deviation as provided here. In this case, the same standard deviation is assumed for all measurements for the given observable.

  Alternatively, some formula expression can be provided to specify more complex noise models. A noise model which accounts for relative and absolute contributions could, e.g., be defined as

  ```
  noiseParameter1_observable_pErk + noiseParameter2_observable_pErk*pErk
  ```

  with `noiseParameter1_observable_pErk` denoting the absolute and `noiseParameter2_observable_pErk` the relative contribution for the observable `observable_pErk` corresponding to species `pErk`. IDs of noise parameters that need to have different values for different measurements have the structure: `noiseParameter${indexOfNoiseParameter}_${observableId}` to facilitate automatic recognition. The specific values or parameters are assigned in the `noiseParameters` field of the *measurement table* (see above). Any parameters named `noiseParameter${1..n}_${observableId}` *must* be overwritten in the measurement table.

- `noiseDistribution` [STRING: 'normal' or 'laplace', OPTIONAL]

  Assumed noise distribution for the given measurement. Only normally or Laplace distributed noise is currently allowed (log-normal and log-laplace are obtained by setting `observableTransformation` to `log`). Defaults to `normal`. If `normal`, the specified `noiseParameters` will be interpreted as standard deviation (*not* variance).

Parameter table

A tab-separated value text file containing information on model parameters.

This table *must* include the following parameters: - Named parameter overrides introduced in the *conditions table*, unless defined in the SBML model - Named parameter overrides introduced in the *measurement table*

and *must not* include: - Placeholder parameters (see `observableParameters` and `noiseParameters` above) - Parameters included as column names in the *condition table* - Parameters that are AssignmentRule targets in the SBML model

it *may* include: - Any SBML model parameter that was not excluded above - Named parameter overrides introduced in the *conditions table*

One row per parameter with arbitrary order of rows and columns:

parameterId	[parameterName]	parameterScale	lowerBound	upperBound	nominalValue	estimate	[priorType]	[priorParameters]
STRING	[STRING]	log10|lin|log	NUMERIC	NUMERIC	NUMERIC	0|1	*see below*	*see below*
...	...	...	...	...	...	...	...	...

Additional columns may be added.

Detailed field description:

- `parameterId` [STRING, NOT NULL]

  The `parameterId` of the parameter described in this row. This has to match the ID of a parameter specified in the SBML model, a parameter introduced as override in the condition table, or a parameter occurring in the `observableParameters` or `noiseParameters` column of the measurement table (see above).

- `parameterName` [STRING, OPTIONAL]

  Parameter name to be used e.g. for plotting etc. Can be chosen freely. May or may not coincide with the SBML parameter name.

- `parameterScale` [lin|log|log10]

  Scale of the parameter to be used during parameter estimation.

- `lowerBound` [NUMERIC]

  Lower bound of the parameter used for optimization. Optional, if `estimate==0`. Must be provided in linear space, independent of `parameterScale`.

- `upperBound` [NUMERIC]

  Upper bound of the parameter used for optimization. Optional, if `estimate==0`. Must be provided in linear space, independent of `parameterScale`.

- `nominalValue` [NUMERIC]

  Some parameter value to be used if the parameter is not subject to estimation (see `estimate` below). Must be provided in linear space, independent of `parameterScale`. Optional, unless `estimate==0`.

- `estimate` [BOOL 0|1]

  1 or 0, depending on, if the parameter is estimated (1) or set to a fixed value(0) (see `nominalValue`).

- `initializationPriorType` [STRING, OPTIONAL]

  Prior types used for sampling of initial points for optimization. Sampled points are clipped to lie inside the parameter boundaries specified by `lowerBound` and `upperBound`. Defaults to `parameterScaleUniform`.

  Possible prior types are:

  - *uniform*: flat prior on linear parameters
  - *normal*: Gaussian prior on linear parameters
  - *laplace*: Laplace prior on linear parameters
  - *logNormal*: exponentiated Gaussian prior on linear parameters
  - *logLaplace*: exponentiated Laplace prior on linear parameters
  - *parameterScaleUniform* (default): Flat prior on original parameter scale (equivalent to "no prior")
  - *parameterScaleNormal*: Gaussian prior on original parameter scale
  - *parameterScaleLaplace*: Laplace prior on original parameter scale

- `initializationPriorParameters` [STRING, OPTIONAL]

  Prior parameters used for sampling of initial points for optimization, separated by a semicolon. Defaults to `lowerBound;upperBound`.

  So far, only numeric values will be supported, no parameter names. Parameters for the different prior types are:

  - uniform: lower bound; upper bound
  - normal: mean; standard deviation (**not** variance)
  - laplace: location; scale
  - logNormal: parameters of corresp. normal distribution (see: normal)
  - logLaplace: parameters of corresp. Laplace distribution (see: laplace)
  - parameterScaleUniform: lower bound; upper bound
  - parameterScaleNormal: mean; standard deviation (**not** variance)

- parameterScaleLaplace: location; scale

- `objectivePriorType` [STRING, OPTIONAL]

  Prior types used for the objective function during optimization or sampling. For possible values, see `initializationPriorType`.

- `objectivePriorParameters` [STRING, OPTIONAL]

  Prior parameters used for the objective function during optimization. For more detailed documentation, see `initializationPriorParameters`.

Visualization table

A tab-separated value file containing the specification of the visualization routines which come with the PEtab repository. Plots are in general collections of different datasets as specified using their `datasetId` (if provided) inside the measurement table.

Expected to have the following columns in any (but preferably this) order:

plotId	[plotName]	[plotTypeSimulation]	[plotTypeData]
STRING	[STRING]	[LinePlot(default)|BarPlot|ScatterPlot]	[MeanAndSD(default)|MeanAndSEM|replicate;provided]
...	...	...	...

(wrapped for readability)

...	[datasetId]	[xValues]	[xOffset]	[xLabel]	[xScale]
...	[datasetId]	[time(default)|parameterOrStateId]	[NUMERIC]	[STRING]	[lin|log|log10|order]
...	...	...	...	...	

(wrapped for readability)

...	[yValues]	[yOffset]	[yLabel]	[yScale]	[legendEntry]
...	[observableId]	[NUMERIC]	[STRING]	[lin|log|log10]	[STRING]
...	...	...	...	...	...

Detailed field description:

- `plotId` [STRING, NOT NULL]

  An ID which corresponds to a specific plot. All datasets with the same plotId will be plotted into the same axes object.

- `plotName` [STRING, OPTIONAL]

  A name for the specific plot.

- `plotTypeSimulation` [STRING, OPTIONAL]

  The type of the corresponding plot, can be `LinePlot`, `BarPlot` and `ScatterPlot`. Default is `LinePlot`.

- `plotTypeData` [STRING, OPTIONAL]

  The type how replicates should be handled, can be `MeanAndSD`, `MeanAndSEM`, `replicate` (for plotting all replicates separately), or `provided` (if numeric values for the noise level are provided in the measurement table). Default is `MeanAndSD`.

- `datasetId` [STRING, NOT NULL, REFERENCES(measurementTable.datasetId), OPTIONAL]

  The datasets which should be grouped into one plot.

- `xValues` [STRING, OPTIONAL]

  The independent variable, which will be plotted on the x-axis. Can be `time` (default, for time resolved data), or it can be `parameterOrStateId` for dose-response plots. The corresponding numeric values will be shown on the x-axis.

- `xOffset` [NUMERIC, OPTIONAL]

  Possible data-offsets for the independent variable (default is `0`).

- `xLabel` [STRING, OPTIONAL]

  Label for the x-axis. Defaults to the entry in `xValues`.

- `xScale` [STRING, OPTIONAL]

  Scale of the independent variable, can be `lin`, `log`, `log10` or `order`. The `order` value should be used if values of the independent variable are ordinal. This value can only be used in combination with `LinePlot` value for the `plotTypeSimulation` column. In this case, points on x axis will be placed equidistantly from each other. Default is `lin`.

- `yValues` [observableId, REFERENCES(measurementTable.observableId), OPTIONAL]

  The observable which should be plotted on the y-axis.

- `yOffset` [NUMERIC, OPTIONAL]

  Possible data-offsets for the observable (default is `0`).

- `yLabel` [STRING, OPTIONAL]

  Label for the y-axis. Defaults to the entry in `yValues`.

- `yScale` [STRING, OPTIONAL]

  Scale of the observable, can be `lin`, `log`, or `log10`. Default is `lin`.

- `legendEntry` [STRING, OPTIONAL]

  The name that should be displayed for the corresponding dataset in the legend and which defaults to the value in `datasetId`.

Extensions

Additional columns, such as `Color`, etc. may be specified.

Examples

Examples of the visualization table can be found in the Benchmark model collection. For example, for Chen_MSB2009 model.

YAML file for grouping files

To link the SBML model, measurement table, condition table, etc. in an unambiguous way, we use a YAML file.

This file also allows specifying a PEtab version (as the format is not unlikely to change in the future).

Furthermore, this can be used to describe parameter estimation problems comprising multiple models (more details below).

The format is described in the schema ../petab/petab_schema.yaml, which allows for easy validation.

Parameter estimation problems combining multiple models

Parameter estimation problems can comprise multiple models. For now, PEtab allows to specify multiple SBML models with corresponding condition and measurement tables, and one joint parameter table. This means that the parameter namespace is global. Therefore, parameters with the same ID in different models will be considered identical.
