# Supplementary material for: PEtab—Interoperable specification of parameter estimation problems in systems biology
Source: PLoS Comput Biol. 2021 Jan 26;17(1):e1008646. doi: 10.1371/journal.pcbi.1008646 (PMC7864467; doi:10.1371/journal.pcbi.1008646)
Supplement: S2 File — Step-by-step instructions for creating PEtab files for an application example. (PDF) [file pcbi.1008646.s002.pdf]

PEtab Tutorial

Contents

Overview **1**

1. The model **1**

2. Linking model and measurements **3**
2.1 Specifying experimental conditions 3
2.2 Specifying the observation model 4
2.3 Specifying measurements 4

3. Defining parameters **5**

4. Visualization file **6**

5. YAML file **6**

6. Model simulation **7**

7. Further information **7**

References **8**

Overview

In the following, we demonstrate how to set up a parameter estimation problem in PEtab based on a realistic application example. To this end, we consider the model and experimental data by Boehm et al. (2014). The model describes the dynamics of phosphorylation and dimerization of the transcription factors STAT5A and STAT5B. A visualization and the corresponding reactions of the model are provided below, although the details of the model are not relevant for the purpose of this tutorial. For more details, we refer to the original publication.

A PEtab problem consists of 1) an SBML model of a biological system, 2) condition, observable and measurement definitions, and 3) the specification of the parameters. We will show how to generate the respective files in the following.

1. The model

PEtab assumes that an SBML file of the model exists. Here, we use the SBML model provided in the original publication, which is also available on Biomodels (https://www.ebi.ac.uk/biomodels/BIOMD0000000591). For illustration purposes we slightly modified

the SBML model and shortened some parts of the PEtab files. The full PEtab problem introduced in this tutorial is available online.

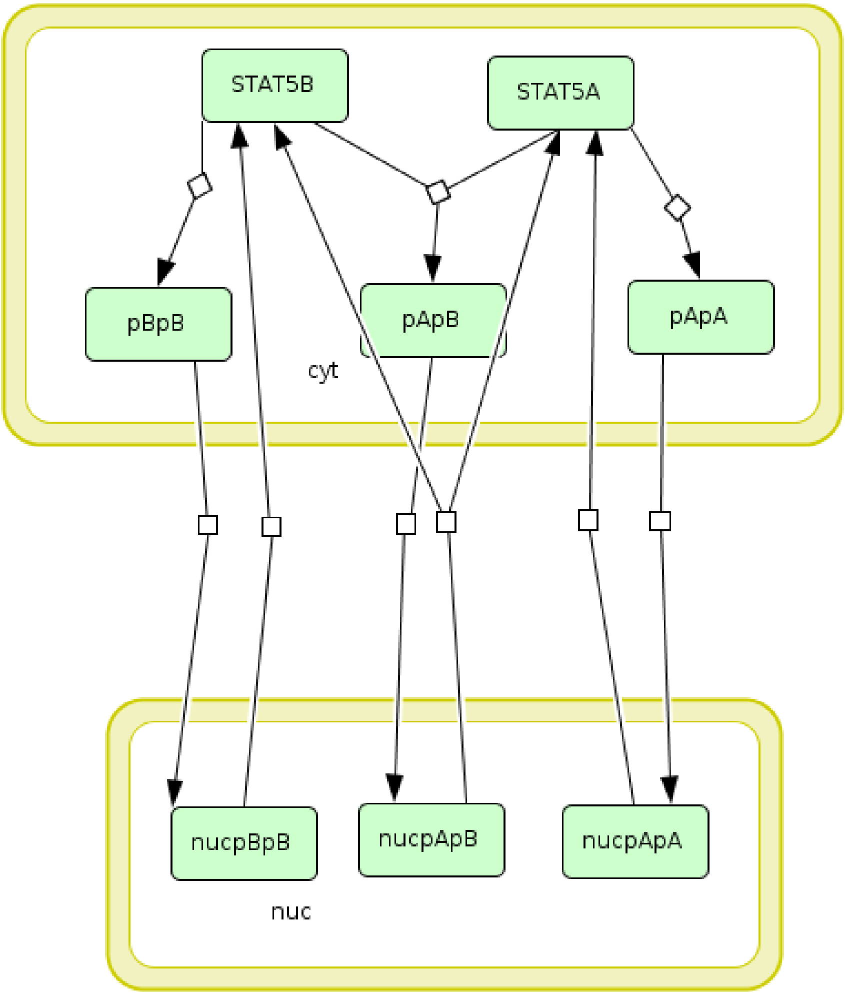

Figure 1: Visualization of the model used as example in this tutorial. The model describes the dynamics of phosphorylation and dimerization of the transcription factors STAT5A and STAT5B.

Table 1: Reactions included in the example model.

ID	Reaction	Rate law
R1	2 STAT5A → pApA	cyt * BaF3_Epo * STAT5A^2 * k_phos
R2	STAT5A + STAT5B → pApB	cyt * BaF3_Epo * STAT5A * STAT5B * k_phos
R3	2 STAT5B → pBpB	cyt * BaF3_Epo * STAT5B^2 * k_phos
R4	pApA → nucpApA	cyt * k_imp_homo * pApA
R5	pApB → nucpApB	cyt * k_imp_hetero * pApB
R6	pBpB → nucpBpB	cyt * k_imp_homo * pBpB
R7	nucpApA → 2 STAT5A	nuc * k_exp_homo * nucpApA

ID	Reaction	Rate law
R8	nucpApB → STAT5A + STAT5B	nuc * k_exp_hetero * nucpApB
R9	nucpBpB → 2 STAT5B	nuc * k_exp_homo * nucpBpB

2. Linking model and measurements

The model by Boehm et al. (2014) was calibrated on measurements on phosphorylation levels of STAT5A and STAT5B as well as relative STAT5A abundance for different timepoints between 0 - 240 minutes after stimulation with erythropoietin (Epo):

To define a parameter estimation problem in PEtab, we need to map measurements to the model states. To this end, we need to 1) specify the experimental conditions the measurements were generated from, 2) specify observation functions and error models, and 3) specify the measurements themselves. For this, we need to define observation functions as well as experimental conditions under which a measurement was performed.

2.1 Specifying experimental conditions

All measurements were collected under the same experimental condition, which is a stimulation with Epo. This is specified in the experimental condition PEtab file, a tab-separated values (TSV) file[1], by providing a condition identifier and listing all condition-specific parameters and their respective values.

In the problem considered here, the relevant parameter is `Epo_concentration` which we want to set to a value of 1.25E-7, as the only condition-specific parameter. Since in this example we include data from only one single experiment, it would not be necessary to specify the condition parameter here, but instead the value could have been also set in the model or in the parameter table. However, the benefit of specifying it in the condition table is, that it allows us to easily add measurements from other experiments performed with different Epo concentrations later on.

The condition table looks as follows:

Table 2: Conditions table `experimental_conditions.tsv`.

conditionId	conditionName	Epo_concentration
epo_stimulation	Stimulation with 1.25E-7 Epo	1.25E-7

- *conditionId* is a unique identifier to define the different conditions and link them to the measurements (see measurement file below). Additional measurements e.g. for different Epo concentrations can be defined by adding new rows.
- *conditionName* can be used as a human readable description of the condition e.g. for plotting.

The following column headers (here *Epo_concentration*) refer to different parameters or species in the model, the values of which are overridden by these condition-specific values. Here, we define the Epo concentration, but additional columns could be used to e.g. set different initial concentrations of STAT5A/B. In addition to numeric values, also parameter identifiers can be used here to introduce condition specific optimization parameters.

[1] TSV files can be created using any standard spreadsheet application, or for small files, text editor.

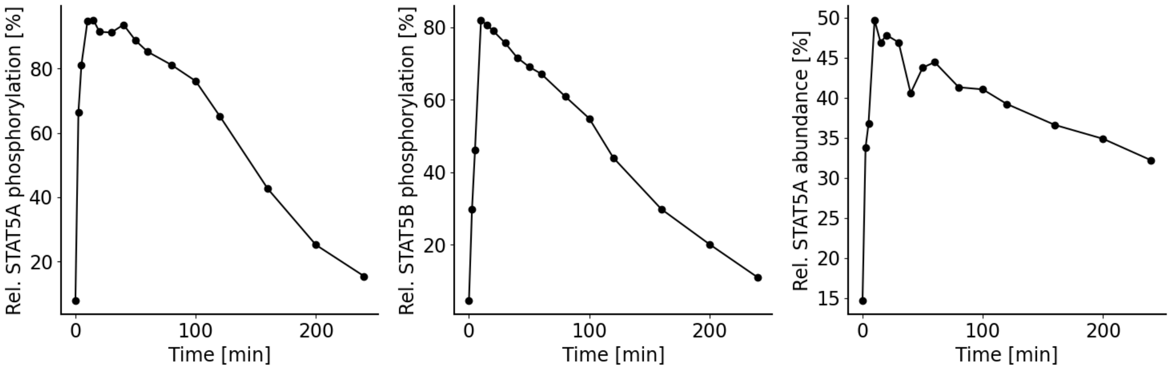

Figure 2: Measurements considered for model calibration in our example.

2.2 Specifying the observation model

To link the model states to the measured values, we specify observation functions. Additionally, a noise model can be introduced to account for the measurement errors. In PEtab, this can be encoded in the observable file:

Table 3: Observables table `observables.tsv`.

observableId	observableName	...
pSTAT5A_rel	Rel. STAT5A phosphorylation [%]	...
pSTAT5B_rel	Rel. STAT5B phosphorylation [%]	...
rSTAT5A_rel	Rel. STAT5A abundance [%]	...

Table 4: Observables table `observables.tsv` (continued).

...	observableFormula	...
...	100*(2*pApA + pApB) / (2*pApA + pApB + STAT5A)	...
...	100*(2*pBpB + pApB) / (2*pBpB + pApB + STAT5B)	...
...	100*(STAT5A + pApB + 2*pApA) / (2 * pApB + 2* pApA + STAT5A + STAT5B + 2*pBpB)	...

Table 5: Observables table `observables.tsv` (continued).

...	noiseFormula	noiseDistribution
...	noiseParameter1_pSTAT5A_rel	normal
...	noiseParameter1_pSTAT5B_rel	normal
...	noiseParameter1_rSTAT5A_rel	normal

- *observableId* specifies a unique identifier to the observables that can be used to link them to the measurements (see below).
- *observableName* can be used as a human readable description of the observable. Here, this corresponds to the y-label in the figure above.
- *observableFormula* is a mathematical expression defining how the model output is calculated. The formula can consist of species and parameters defined in the SBML file. In our example, we measure e.g. the relative phosphorylation level of STAT5A (*pSTAT5A_rel*), which is the sum of all species containing phosphorylated STAT5A over the sum of all species containing any form of STAT5A.
- *noiseFormula* is used to describe the formula for the measurement noise. Together with *noiseDistribution*, it defines the noise model. In this example, we assume additive, normally distributed measurement noise. In this scenario, `noiseParameter1_{observableId}` is the standard deviation of the measurement noise. Parameters following this naming scheme are expected to be overridden in a measurement-specific manner in the *noiseParameters* column of the measurement

Table 6: Measurement table `measurement_data.tsv`.

observableId	simulationConditionId	measurement	time	noiseParameters
pSTAT5A_rel	epo_stimulation	7.9	0	sd_pSTAT5A_rel
...	...	...	...	...
pSTAT5A_rel	epo_stimulation	15.4	240	sd_pSTAT5A_rel
pSTAT5B_rel	epo_stimulation	4.6	0	sd_pSTAT5B_rel
...	...	...	...	...
pSTAT5B_rel	epo_stimulation	10.96	240	sd_pSTAT5B_rel
rSTAT5A_rel	epo_stimulation	14.7	0	sd_rSTAT5A_rel
...	...	...	...	...
rSTAT5A_rel	epo_stimulation	32.2	240	sd_rSTAT5A_rel

- *observableId* references the *observableId* from the observable file.
- *simulationConditionId* references the *conditionId* from the experimental condition file.
- *measurement* defines the values that are measured for the respective observable and experimental condition.
- *time* is the time point at which the measurement was performed. For brevity, only the first and last time point of the example are shown here (the omitted measurements are indicated by "..." in the example).
- *noiseParameters* relates to the *noiseParameters* in the observables file. In our example, the measurement noise is unknown. Therefore we define parameters here which have to be estimated (see parameters sheet below). If the noise is known, e.g. from multiple replicates, numeric values can be used in this column.

3. Defining parameters

The model by Boehm et al. (2014) contains nine unknown parameters that need to be estimated from the experimental data. Additionally, it has two known parameters that are fixed to literature values.

The parameters file for this is given by:

Table 7: Parameter table `parameters.tsv`.

parameterId	parameterScale	lowerBound	upperBound	nominalValue	estimate
Epo_degradation_BaF3	log10	1E-5	1E+5		1
k_exp_hetero	log10	1E-5	1E+5		1
k_exp_homo	log10	1E-5	1E+5		1
k_imp_hetero	log10	1E-5	1E+5		1
k_imp_homo	log10	1E-5	1E+5		1
k_phos	log10	1E-5	1E+5		1
ratio	lin			0.693	0
sd_pSTAT5A_rel	log10	1E-5	1E+5		1
sd_pSTAT5B_rel	log10	1E-5	1E+5		1
sd_rSTAT5A_rel	log10	1E-5	1E+5		1

- *parameterId* references parameters defined in the SBML file. Additionally, parameters defined in the measurement table can be used here. In this example, the

standard deviations for the different observables (*sd_{observableId}*) are estimated.

- *parameterScale* is the scale on which parameters are estimated. Often, a logarithmic scale improves optimization. Alternatively, a linear scale can be used, e.g. when parameters can be negative.
- *lowerBound* and *upperBound* define the bounds for the parameters used during optimization. These are usually biologically plausible ranges.
- *nominalValue* are known values used for simulation. The entry can be left empty, if a value is unknown and subject to optimization.
- *estimate* defines whether the parameter is subject to optimization (1) or if it is fixed (0) to the value in the nominalValue column.

4. Visualization file

Optionally, a visualization file can be specified in PEtab which defines how the measurement data and potentially model simulations are plotted. So far, the visualization files are only supported by the PEtab Python library. Here, we describe a file that specifies the visualization of the measurement data similar to the figure above.

Table 8: Visualization specification table `visualization_specification.tsv`.

plotId	plotTypeData	xLabel	yValues	yLabel
plot1	MeanAndSD	Time [min]	pSTAT5A_rel	Rel. STAT5A phosphorylation [%]
plot2	MeanAndSD	Time [min]	pSTAT5B_rel	Rel. STAT5B phosphorylation [%]
plot3	MeanAndSD	Time [min]	rSTAT5A_rel	Rel. STAT5A abundance [%]

- *plotId* corresponds to a specific plot. All lines which share the same *plotId* are combined into one plot.
- *plotTypeData* defines the plotting style of the measurement data. Here, we use mean and (if available) standard deviations.
- *xLabel* and *yLabel* are the labels of the x- and y-axes for the corresponding plot.
- *yValues* defines what is plotted. In this example the different observables are plotted individually.

There are various ways of further individualizing the plots, e.g. by defining legend entries or data plotted on log-scale (see the documentation for further information https://petab.readthedocs.io/en/latest/documentation_data_format.html#visualization-table).

5. YAML file

To group the previously mentioned PEtab files, a YAML file can be used, defining which files constitute a PEtab problem. While being optional, this makes it easier to import a PEtab problem into tools, and allows reusing files for different PEtab problems. This file has the following format (`Boehm_JProteomeRes2014.yaml`):

```yaml
format_version: 1
parameter_file: parameters.tsv
problems:
  - condition_files:
    - experimental_conditions.tsv
    measurement_files:
```

```
- measurement_data.tsv
observable_files:
- observables.tsv
sbml_files:
- model_Boehm_JProteomeRes2014.xml
visualization_files:
- visualization_specification.tsv
```

The first line specifies the version this file and the files referenced adhere to. The current version number is 1. The second line references the parameter file. This is followed by a list of (sub-)problems, in this case only one, referencing the respective condition, measurement observable, model, and visualization files. There can be multiple of those files, e.g. for large numbers of measurements, one could split those up into separate files, e.g. by experimental condition or observable.

6. Model simulation

To simulate the model and compare it to the experimental data, the nominal parameters in the parameters file need to be set. As some parameters are a priori unknown, we here consider randomly sampled parameters to get a glance of model behaviour and fit to the data.

Table 9: Parameter table `parameters.tsv` with *nominalValue* set to random values.

parameterId	parameterScale	lowerBound	upperBound	nominalValue	estimate
Epo_degradation_BaF3	log10	1E-5	1E+5	0.105	1
k_exp_hetero	log10	1E-5	1E+5	1.85	1
k_exp_homo	log10	1E-5	1E+5	9.83	1
k_imp_hetero	log10	1E-5	1E+5	1048.96	1
k_imp_homo	log10	1E-5	1E+5	10.136	1
k_phos	log10	1E-5	1E+5	10.136	1
ratio	lin			0.693	0
sd_pSTAT5A_rel	log10	1E-5	1E+5	51.7	1
sd_pSTAT5B_rel	log10	1E-5	1E+5	0.257	1
sd_rSTAT5A_rel	log10	1E-5	1E+5	0.017	1

With this, the model can be simulated using the different tools that support PEtab. The easiest tool to get started with is probably COPASI which comes with a graphical user interface (see https://github.com/copasi/python-petab-importer for further instructions).

It is apparent from the figure, that the random parameters yield a poor fit of the model with the data. Therefore, it is important to optimize the parameters to improve the model fit. This can be done using various parameter estimation tools. Links to detailed descriptions how to use the individual toolboxes are provided at the PEtab Github page.

7. Further information

This tutorial only demonstrates a subset of PEtab functionality. For full reference, consult the PEtab reference. After finishing the implementation of the PEtab problem,

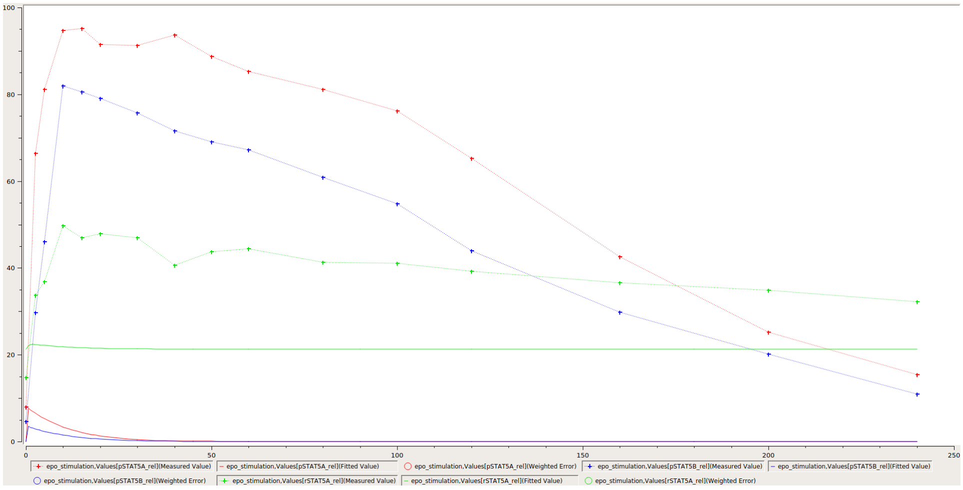

Figure 3: Visualization of model outputs after simulation with random parameters and measurements in COPASI.

its correctness can be verified using the PEtab library (see https://github.com/PEtab-dev/PEtab/blob/master/doc/example/example_petablint.ipynb for instructions). The PEtab problem can then be used as input to the supporting toolboxes to estimate the unknown parameters or calculate parameter uncertainties. Links to tutorials for the different tools can be found at the PEtab Github page (https://github.com/PEtab-dev/PEtab#petab-support-in-systems-biology-tools).

References

- Martin E. Boehm, Lorenz Adlung, Marcel Schilling, Susanne Roth, Ursula Klingmüller, and Wolf D. Lehmann. *Journal of Proteome Research* **2014** 13 (12), 5685-5694. DOI: 10.1021/pr5006923.
