# Supplementary material for: PEtab—Interoperable specification of parameter estimation problems in systems biology
Source: PLoS Comput Biol. 2021 Jan 26;17(1):e1008646. doi: 10.1371/journal.pcbi.1008646 (PMC7864467; doi:10.1371/journal.pcbi.1008646)
Supplement: Attachment — Submitted filename: PEtab_response_letter.pdf [file pcbi.1008646.s003.pdf]

Author's response to editor's and reviewers' comments

We thank the editor and the reviewers for their constructive comments. Based on their remarks, we have revised the manuscript. We addressed all their comments and tried to resolve the raised issues. Our point-by-point response is provided below.

The teal boxes contain the editor's and the reviewers' comments, while the yellow boxes contain the changes made in the manuscript. To provide some context, the changes (blue) are surrounded by unchanged text (black).

Response to Editor

Thank you very much for submitting your manuscript "PEtab – Interoperable Specification of Parameter Estimation Problems in Systems Biology" for consideration at PLOS Computational Biology. As with all papers reviewed by the journal, your manuscript was reviewed by members of the editorial board and by several independent reviewers. The reviewers appreciated the attention to an important topic. Based on the reviews, we are likely to accept this manuscript for publication, providing that you modify the manuscript according to the review recommendations.
The reviewers were positive about the PEtab format, however it will be useful to include in the manuscript examples, as suggested by reviewer 1. A basic and more advanced example of PEtab can help potential users.

Dear Dina Schneidman,

thank you for the positive feedback. We revised the manuscript according to the reviewers' comments. Most importantly, we now provide a detailed step-by-step example for setting up a PEtab problem. The example demonstrates basic features such as specifying the observation model and measurements, but also more advanced features such as condition- and measurement-specific parameter overriding.

In the following, we provide a detailed point-by-point response to the reviewer's comments.

Response to Reviewer 1

This manuscript is a report about a new standardized data format called PEtab. The data represented in the PEtab format is that needed to solve curve fitting (or model parametrization) problems. Examples of PEtab files (or rather collections of files) are provided at the GitHub repository cited in the manuscript. The format is supported by various software tools developed to support systems biology modeling. Importantly, these tools include useful ones, and moreover, the collection offers an array of non-overlapping capabilities. The description of PEtab is probably sufficiently detailed for those who may wish to adopt the format (as a user or developer of a new or existing software package). PEtab is potentially useful in that it could make it easier for modelers to leverage multiple software packages, because problem setup should be at least similar now for all of the tools supporting PEtab. To my best knowledge, there is no standardized format for the definition of curve-fitting problems in the systems biology modeling field.

We appreciate that the reviewer acknowledges the significance of our work.

A concern is potential impact. The authors have not provided a compelling demonstration of the benefit of having a standardized format. Time will tell if the format is adopted and proves to be helpful in moving the field of systems biology modeling forward.

We agree with the reviewer in that it is hard to anticipate the impact of a new standard such as PEtab. We think that nobody would have expected the impact that standards like SBML and SBGN had on the field of systems biology. We think that PEtab can add to existing standards as it is clear that there is a need for some kind of standardization of parameter estimation problems in the field.

A potential impediment for the adoption of developed standards is lack of tool support and thereby lack of benefits to the user. Therefore, we initially implemented PEtab support to 8 different tools. Meanwhile another tool has been developed that implements PEtab support by a non-co-author (https://github.com/paulflang/SBML2Julia). The benefit for the user should come from the fact that, as the reviewer noted in his previous comment, "the collection [of tools supporting PEtab] offers an array of non-overlapping capabilities", which are now much easier to exploit. Furthermore, PEtab should improve reusability and reproducibility.

Changes in the manuscript:

Initial submission: [...] We encourage additional toolbox developers to implement support for PEtab.

Revised submission: [...] We encourage additional toolbox developers to implement support for PEtab. As an example, since the preprint publication of this manuscript, PEtab has already been adopted as the input format for a newly developed tool, SBML2Julia (26).

Another concern is that the manuscript lacks an accessible introduction to PEtab for the new user. It would be nice if the authors could add a step-by-step problem-setup guide that walks the reader through setup of one or more simple fitting problems using PEtab from start to finish. I think this addition to the text would greatly improve accessibility of the format. The guidance provided in the manuscript at present is of a general nature, and problem setup seems to be somewhat complicated. Consideration of a specific demonstration problem (or a few of different kinds, ideally) would make the guidance on using the new format more concrete.

We thank the reviewer for bringing up the important topic of easy accessibility for new users.

We created a tutorial based on a realistic application example to introduce the main features of PEtab. Due to the necessary level of detail and the resulting length of the tutorial including it in the main manuscript seems problematic. Therefore, we added the tutorial as Supplementary Information and included it in the online documentation.

Changes in the manuscript:

Initial submission: -

Revised submission: Additionally, we created a tutorial illustrating how to set up a PEtab problem, covering the most common features (supplementary file S2 File).

Changes in the manuscript:

Initial submission: -

Revised submission: [Supplementary file S2 is included at the end of this file for readability.]

Response to Reviewer 2

PEtab is a data format for specifying systems biology parameter estimation problems based on Tab-Separated Values (TSV) files. Currently, PEtab only allows for models defined in SBML format and is supported by eight systems biology toolboxes, with long term support facilitated by a maintainer team comprising developers from these toolboxes. Parameter estimation problems are specified by an SBML model specifying the biological process under investigation and the following TSV files:
a. A measurement file to fit the model to
b. A condition file specifying model inputs and condition-specific parameters
c. An observable file specifying the observation model
d. A parameter file specifying optimization parameters and related information
e. (optional) A simulation file, which has the same format as the measurement file, but contains model simulations
f. (optional) A visualization file, which contains specifications how the data and/or simulations should be plotted by the visualization routines
Additionally a PEtab problem file, written in YAML format (currently 1.2), is needed to link all of the afore-mentioned PEtab files. Links to Github repositories with detailed documentations describing the formats of each file as well as installation instructions for the python library developed to create, check, visualize and work with PEtab files are provided in the manuscript. A wide range of system biology parameter estimation PEtab examples are also provided as benchmark problems. The authors acknowledge the existing tools which are used for simulating models and inferring parameters and highlight the community standards used by these tools. The authors do well to highlight the importance of their tool by describing the lack of interoperability between current parameter estimation tools and the community based standards. PEtab represents an excellent attempt to standardize parameter estimation problems in Systems Biology and will greatly improve the reusability, reproducibility and interoperability of such models in this domain.

We thank the reviewer for appreciating the relevance of the PEtab format.

However further integration with other model- and rule-based formats is needed, as although inter-standard converters exist and can potentially convert these formats into SBML, they are known to be prone to errors and are not smoothly implemented.

We agree with the reviewer, that lossless interconversion of model formats is still an open problem. We think that this is a problem worth being addressed independently of our standardization efforts here. At the same time, we agree, that PEtab would appeal to more users, if allowing for additional model formats. This is an issue we want to address in the next version of PEtab. In fact, there is already a draft implementation for supporting (rule-based) PySB models with PEtab in AMICI and pyPESTO. As PySB allows for the import of various other rule-based modelling formats, e.g. BioNetGen Language (BNGL) or Kappa, PEtab is in principle already applicable here. We envisage a high degree of flexibility for model formats, additionally covering, for example, CellML and BNGL. However, as this will require further discussions and implementation work, we consider this extension beyond the scope of the current manuscript.

Minor Comments:
PEtab problem specification format: *"A detailed specification of PEtab version 1 is provided in supplementary file S1 File, as well as at https://github.com/PEtab-dev/PEtab. Further example problems can be found at https://github.com/Benchmarking-Initiative/Benchmark-Models-PEtab. The different files specify the biological model, the observation model, experimental conditions, measurements, parameters and visualizations (Fig 1B)."*
The supplementary file and links to the Github repository for the source code and benchmarking implementations are most welcomed. I was able to run the bench-marking models on multiple supported platforms, further confirming the interoperability of the data format developed.

We appreciate the reviewer's extra efforts for actually trying out the tools and examples.

However I suggest adding a figure similar to Fig1B in the manuscript to the Github documentation page for further clarification and representation of the workflow elements in PEtab.

Thanks for the suggestion. We added the complete Figure 1 to the online documentation.

"Parameter hierarchy and pre-equilibrium in PEtab Fig 3 A and B": The caption and graphical representation in Figure 3A needs further clarity. I believe that the authors are describing the ability to override parameters through the use of measurements or conditions accounting for observational parameters or model inputs but this is not entirely clear from the figures.

The reviewers' interpretation of Figure 3A was absolutely correct. We adapted the illustration as well as the caption to clarify the parameter overriding.

Changes in the manuscript:

Initial submission:

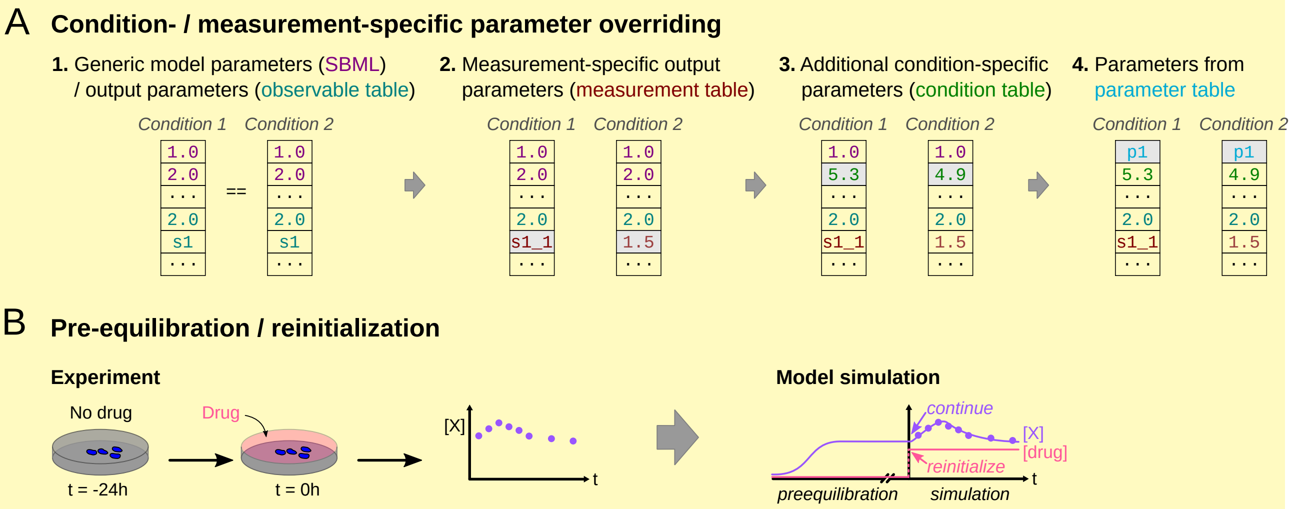

Parameter hierarchy and pre-equilibration in PEtab. (A) Illustration of possibilities and precedence of parameter overriding at different stages. Simulation parameter vectors are shown for two simulation conditions. A generic model parameterization can be overridden in a condition- and measurement-specific manner to account for different model inputs or observational model parameters. Thick boxes indicates the newly overridden parameters in each step. Individual parameters can be set to specific values or marked to be estimated (as here p1). (B) In an often encountered experimental setup, a biological system is under some "baseline" condition and assumed to be in equilibrium (e.g., here depicted for after 24h incubation) before a perturbation is applied. If the equilibrium state of the system is not known a priori, such a setup can be modeled by simulating the system until an apparent steady-state is reached (pre-equilibration). To simulate the perturbation, a subset of model states are reinitialized.

Revised submission:

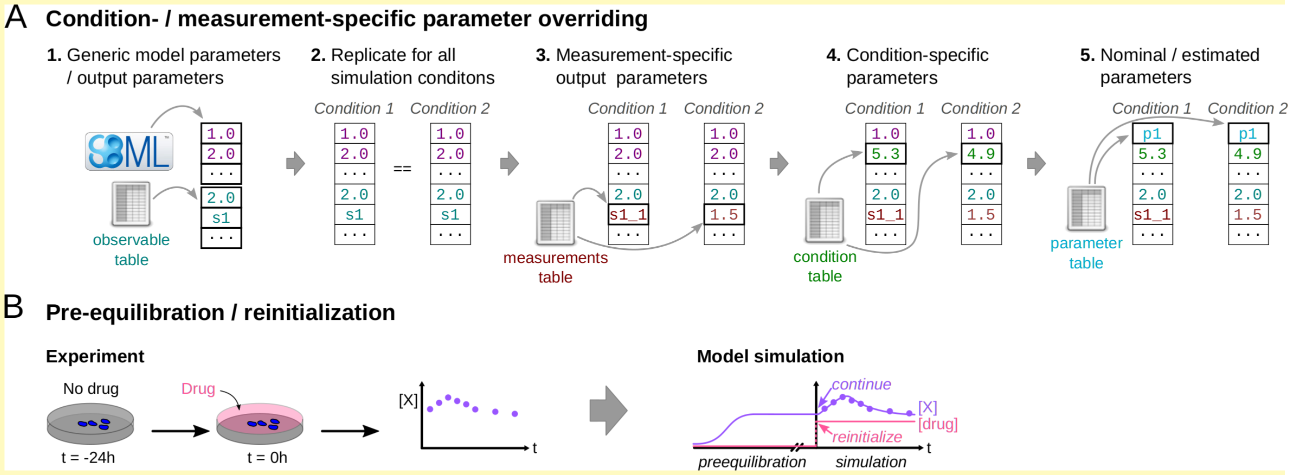

Parameter hierarchy and pre-equilibration in PEtab. (A) Illustration of possibilities and precedence of parameter overriding at different stages. The generic model parameter vector, as specified in the SBML model, can be overridden via the observable, measurement, condition and parameter tables, differentially for conditions and measurement points to account for different model inputs or observational model parameters. The parameters that are overridden in each step are indicated with thicker cell borders. Individual parameters can be set to specific values or marked to be estimated (as here p1). (B) In an often encountered experimental setup, a biological system is under some "baseline" condition and assumed to be in equilibrium (e.g., here depicted for after 24h incubation) before a perturbation is applied. If the equilibrium state of the system is not known a priori, such a setup can be modeled by simulating the system until an apparent steady-state is reached (pre-equilibration). To simulate the perturbation, a subset of model states are reinitialized.

"We formed a maintainer team comprising developers of all supporting toolboxes to facilitate long-term support and improvement of PEtab." This is an excellent initiative by the authors and tool developers. Perhaps a general point of contact to disseminate queries to the specific tool developers should be mentioned. Perhaps through the github page or similar.

We added the following sentence, pointing to the respective GitHub pages for any questions or requests, as suggested:

Changes in the manuscript:

Initial submission: -

Revised submission: For any questions or requests related to PEtab, we encourage interested users to approach us via the Issues function in the aforementioned GitHub repository, or the respective tool repositories for more specific queries.

Newly added supplementary file 2

PEtab Tutorial

Contents

Overview **1**

1. The model **1**

2. Linking model and measurements **3**
2.1 Specifying experimental conditions 3
2.2 Specifying the observation model 4
2.3 Specifying measurements 5

3. Defining parameters **6**

4. Visualization file **6**

5. YAML file **7**

6. Model simulation **7**

7. Further information **8**

Overview

In the following, we demonstrate how to set up a parameter estimation problem in PEtab based on a realistic application example. To this end, we consider the model and experimental data by Boehm et al. (2014). The model describes the dynamics of phosphorylation and dimerization of the transcription factors STAT5A and STAT5B. A visualization and the corresponding reactions of the model are provided below, although the details of the model are not relevant for the purpose of this tutorial. For more details, we refer to the original publication (Boehm et al., 2014).

A PEtab problem consists of 1) an SBML model of a biological system, 2) condition, observable and measurement definitions, and 3) the specification of the parameters. We will show how to generate the respective files in the following.

1. The model

PEtab assumes that an SBML file of the model exists. Here, we use the SBML model provided in the original publication, which is also available on Biomodels (https://www.ebi.ac.uk/biomodels/BIOMD0000000591). For illustration purposes we slightly modified the SBML model and shortened some parts of the PEtab files. The full PEtab problem introduced in this tutorial is available online.

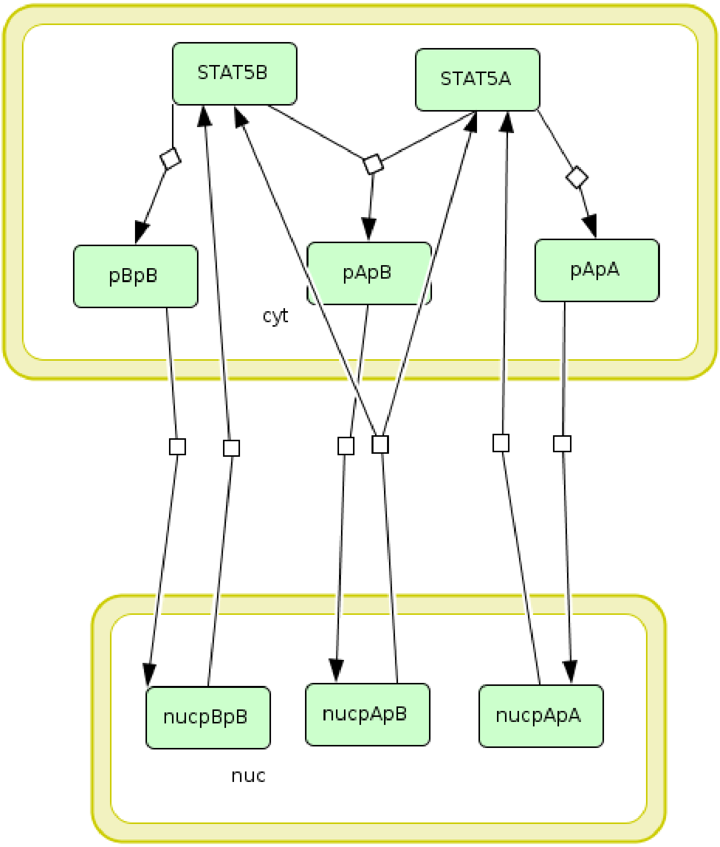

Figure 1: Visualization of the model used as example in this tutorial. The model describes the dynamics of phosphorylation and dimerization of the transcription factors STAT5A and STAT5B.

Table 1: Reactions included in the example model.

ID	Reaction	Rate law
R1	2 STAT5A → pApA	cyt * BaF3_Epo * STAT5A^2 * k_phos
R2	STAT5A + STAT5B → pApB	cyt * BaF3_Epo * STAT5A * STAT5B * k_phos
R3	2 STAT5B → pBpB	cyt * BaF3_Epo * STAT5B^2 * k_phos
R4	pApA → nucpApA	cyt * k_imp_homo * pApA
R5	pApB → nucpApB	cyt * k_imp_hetero * pApB
R6	pBpB → nucpBpB	cyt * k_imp_homo * pBpB
R7	nucpApA → 2 STAT5A	nuc * k_exp_homo * nucpApA
R8	nucpApB → STAT5A + STAT5B	nuc * k_exp_hetero * nucpApB
R9	nucpBpB → 2 STAT5B	nuc * k_exp_homo * nucpBpB

2. Linking model and measurements

The model by Boehm et al. (2014) was calibrated on measurements on phosphorylation levels of STAT5A and STAT5B as well as relative STAT5A abundance for different timepoints between 0 - 240 minutes after stimulation with erythropoietin (Epo):

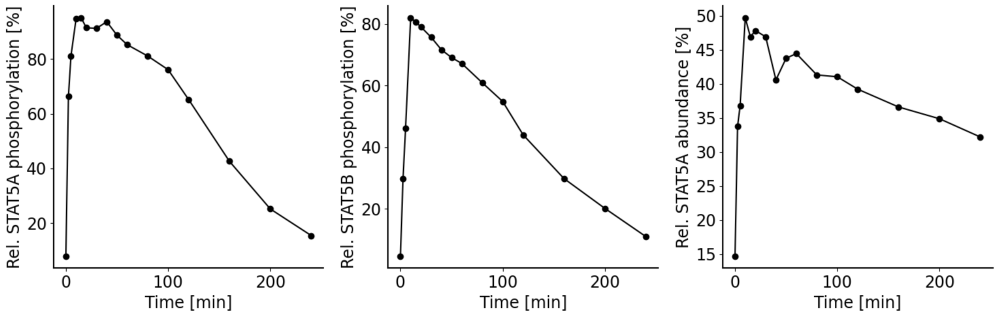

Figure 2: Measurements considered for model calibration in our example.

To define a parameter estimation problem in PEtab, we need to map measurements to the model states. To this end, we need to 1) specify the experimental conditions the measurements were generated from, 2) specify observation functions and error models, and 3) specify the measurements themselves. For this, we need to define observation functions as well as experimental conditions under which a measurement was performed.

2.1 Specifying experimental conditions

All measurements were collected under the same experimental condition, which is a stimulation with Epo. This is specified in the experimental condition PEtab file, a tab-separated values (TSV) file[1], by providing a condition identifier and listing all condition-specific parameters and their respective values.

In the problem considered here, the relevant parameter is `Epo_concentration` which we want to set to a value of 1.25E-7, as the only condition-specific parameter. Since in

[1]TSV files can be created using any standard spreadsheet application, or for small files, text editor.

this example we include data from only one single experiment, it would not be necessary to specify the condition parameter here, but instead the value could have been also set in the model or in the parameter table. However, the benefit of specifying it in the condition table is, that it allows us to easily add measurements from other experiments performed with different Epo concentrations later on.

The condition table looks as follows:

Table 2: Conditions table `experimental_conditions.tsv`.

conditionId	conditionName	Epo_concentration
epo_stimulation	Stimulation with 1.25E-7 Epo	1.25E-7

- *conditionId* is a unique identifier to define the different conditions and link them to the measurements (see measurement file below). Additional measurements e.g. for different Epo concentrations can be defined by adding new rows.
- *conditionName* can be used as a human readable description of the condition e.g. for plotting.

The following column headers (here *Epo_concentration*) refer to different parameters or species in the model, the values of which are overridden by these condition-specific values. Here, we define the Epo concentration, but additional columns could be used to e.g. set different initial concentrations of STAT5A/B. In addition to numeric values, also parameter identifiers can be used here to introduce condition specific optimization parameters.

2.2 Specifying the observation model

To link the model states to the measured values, we specify observation functions. Additionally, a noise model can be introduced to account for the measurement errors. In PEtab, this can be encoded in the observable file:

Table 3: Observables table `observables.tsv`.

observableId	observableName	...
pSTAT5A_rel	Rel. STAT5A phosphorylation [%]	...
pSTAT5B_rel	Rel. STAT5B phosphorylation [%]	...
rSTAT5A_rel	Rel. STAT5A abundance [%]	...

Table 4: Observables table `observables.tsv` (continued).

...	observableFormula	...
...	100*(2*pApA + pApB) / (2*pApA + pApB + STAT5A)	...
...	100*(2*pBpB + pApB) / (2*pBpB + pApB + STAT5B)	...
...	100*(STAT5A + pApB + 2*pApA) / (2 * pApB + 2* pApA + STAT5A + STAT5B + 2*pBpB)	...

Table 5: Observables table `observables.tsv` (continued).

...	noiseFormula	noiseDistribution
...	noiseParameter1_pSTAT5A_rel	normal
...	noiseParameter1_pSTAT5B_rel	normal
...	noiseParameter1_rSTAT5A_rel	normal

- *observableId* specifies a unique identifier to the observables that can be used to link them to the measurements (see below).
- *observableName* can be used as a human readable description of the observable. Here, this corresponds to the y-label in the figure above.
- *observableFormula* is a mathematical expression defining how the model output is calculated. The formula can consist of species and parameters defined in the SBML file. In our example, we measure e.g. the relative phosphorylation level of STAT5A (*pSTAT5A_rel*), which is the sum of all species containing phosphorylated STAT5A over the sum of all species containing any form of STAT5A.
- *noiseFormula* is used to describe the formula for the measurement noise. Together with *noiseDistribution*, it defines the noise model. In this example, we assume additive, normally distributed measurement noise. In this scenario, `noiseParameter1_{observableId}` is the standard deviation of the measurement noise. Parameters following this naming scheme are expected to be overridden in a measurement-specific manner in the *noiseParameters* column of the measurement table (see below).

2.3 Specifying measurements

The experimental data is linked to the conditions via the *conditionId* and to the observables via the *observableId*. This is defined in the PEtab measurement file:

Table 6: Measurement table `measurement_data.tsv`.

observableId	simulationConditionId	measurement	time	noiseParameters
pSTAT5A_rel	epo_stimulation	7.9	0	sd_pSTAT5A_rel
...	...	...	...	...
pSTAT5A_rel	epo_stimulation	15.4	240	sd_pSTAT5A_rel
pSTAT5B_rel	epo_stimulation	4.6	0	sd_pSTAT5B_rel
...	...	...	...	...
pSTAT5B_rel	epo_stimulation	10.96	240	sd_pSTAT5B_rel
rSTAT5A_rel	epo_stimulation	14.7	0	sd_rSTAT5A_rel
...	...	...	...	...
rSTAT5A_rel	epo_stimulation	32.2	240	sd_rSTAT5A_rel

- *observableId* references the *observableId* from the observable file.
- *simulationConditionId* references the *conditionId* from the experimental condition file.
- *measurement* defines the values that are measured for the respective observable and experimental condition.
- *time* is the time point at which the measurement was performed. For brevity, only the first and last time point of the example are shown here (the omitted measurements are indicated by "..." in the example).
- *noiseParameters* relates to the *noiseParameters* in the observables file. In our

example, the measurement noise is unknown. Therefore we define parameters here which have to be estimated (see parameters sheet below). If the noise is known, e.g. from multiple replicates, numeric values can be used in this column.

3. Defining parameters

The model by Boehm et al. (2014) contains nine unknown parameters that need to be estimated from the experimental data. Additionally, it has two known parameters that are fixed to literature values.

The parameters file for this is given by:

Table 7: Parameter table `parameters.tsv`.

parameterId	parameterScale	lowerBound	upperBound	nominalValue	estimate
Epo_degradation_BaF3	log10	1E-5	1E+5		1
k_exp_hetero	log10	1E-5	1E+5		1
k_exp_homo	log10	1E-5	1E+5		1
k_imp_hetero	log10	1E-5	1E+5		1
k_imp_homo	log10	1E-5	1E+5		1
k_phos	log10	1E-5	1E+5		1
ratio	lin			0.693	0
sd_pSTAT5A_rel	log10	1E-5	1E+5		1
sd_pSTAT5B_rel	log10	1E-5	1E+5		1
sd_rSTAT5A_rel	log10	1E-5	1E+5		1

- *parameterId* references parameters defined in the SBML file. Additionally, parameters defined in the measurement table can be used here. In this example, the standard deviations for the different observables (*sd_{observableId}*) are estimated.
- *parameterScale* is the scale on which parameters are estimated. Often, a logarithmic scale improves optimization. Alternatively, a linear scale can be used, e.g. when parameters can be negative.
- *lowerBound* and *upperBound* define the bounds for the parameters used during optimization. These are usually biologically plausible ranges.
- *nominalValue* are known values used for simulation. The entry can be left empty, if a value is unknown and subject to optimization.
- *estimate* defines whether the parameter is subject to optimization (1) or if it is fixed (0) to the value in the nominalValue column.

4. Visualization file

Optionally, a visualization file can be specified in PEtab which defines how the measurement data and potentially model simulations are plotted. So far, the visualization files are only supported by the PEtab Python library. Here, we describe a file that specifies the visualization of the measurement data similar to the figure above.

Table 8: Visualization specification table `visualization_specification.tsv`.

plotId	plotTypeData	xLabel	yValues	yLabel
plot1	MeanAndSD	Time [min]	pSTAT5A_rel	Rel. STAT5A phosphorylation [%]
plot2	MeanAndSD	Time [min]	pSTAT5B_rel	Rel. STAT5B phosphorylation [%]
plot3	MeanAndSD	Time [min]	rSTAT5A_rel	Rel. STAT5A abundance [%]

- *plotId* corresponds to a specific plot. All lines which share the same *plotId* are combined into one plot.
- *plotTypeData* defines the plotting style of the measurement data. Here, we use mean and (if available) standard deviations.
- *xLabel* and *yLabel* are the labels of the x- and y-axes for the corresponding plot.
- *yValues* defines what is plotted. In this example the different observables are plotted individually.

There are various ways of further individualizing the plots, e.g. by defining legend entries or data plotted on log-scale (see the documentation for further information https://petab.readthedocs.io/en/latest/documentation_data_format.html#visualization-table).

5. YAML file

To group the previously mentioned PEtab files, a YAML file can be used, defining which files constitute a PEtab problem. While being optional, this makes it easier to import a PEtab problem into tools, and allows reusing files for different PEtab problems. This file has the following format (`Boehm_JProteomeRes2014.yaml`):

```yaml
format_version: 1
parameter_file: parameters.tsv
problems:
  - condition_files:
    - experimental_conditions.tsv
    measurement_files:
    - measurement_data.tsv
    observable_files:
    - observables.tsv
    sbml_files:
    - model_Boehm_JProteomeRes2014.xml
    visualization_files:
    - visualization_specification.tsv
```

The first line specifies the version this file and the files referenced adhere to. The current version number is 1. The second line references the parameter file. This is followed by a list of (sub-)problems, in this case only one, referencing the respective condition, measurement observable, model, and visualization files. There can be multiple of those files, e.g. for large numbers of measurements, one could split those up into separate files, e.g. by experimental condition or observable.

6. Model simulation

To simulate the model and compare it to the experimental data, the nominal parameters in the parameters file need to be set. As some parameters are a priori unknown, we here

consider randomly sampled parameters to get a glance of model behaviour and fit to the data.

Table 9: Parameter table `parameters.tsv` with *nominalValue* set to random values.

parameterId	parameterScale	lowerBound	upperBound	nominalValue	estimate
Epo_degradation_BaF3	log10	1E-5	1E+5	0.105	1
k_exp_hetero	log10	1E-5	1E+5	1.85	1
k_exp_homo	log10	1E-5	1E+5	9.83	1
k_imp_hetero	log10	1E-5	1E+5	1048.96	1
k_imp_homo	log10	1E-5	1E+5	10.136	1
k_phos	log10	1E-5	1E+5	10.136	1
ratio	lin			0.693	0
sd_pSTAT5A_rel	log10	1E-5	1E+5	51.7	1
sd_pSTAT5B_rel	log10	1E-5	1E+5	0.257	1
sd_rSTAT5A_rel	log10	1E-5	1E+5	0.017	1

With this, the model can be simulated using the different tools that support PEtab. The easiest tool to get started with is probably COPASI which comes with a graphical user interface (see https://github.com/copasi/python-petab-importer for further instructions).

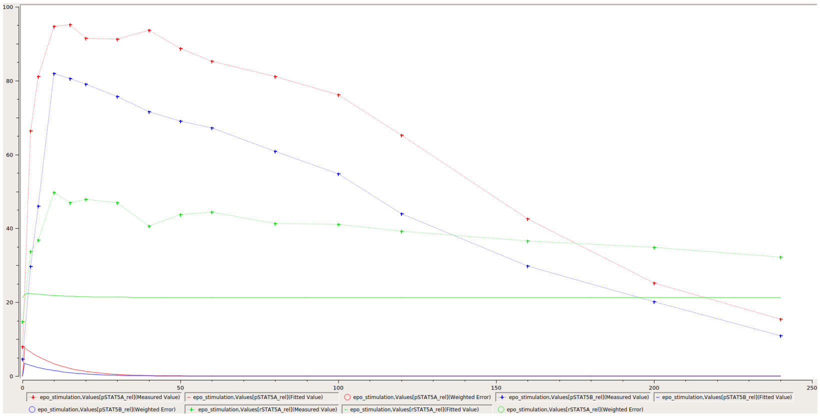

Figure 3: Visualization of model outputs after simulation with random parameters and measurements in COPASI.

It is apparent from the figure, that the random parameters yield a poor fit of the model with the data. Therefore, it is important to optimize the parameters to improve the model fit. This can be done using various parameter estimation tools. Links to detailed descriptions how to use the individual toolboxes are provided at the PEtab Github page.

7. Further information

This tutorial only demonstrates a subset of PEtab functionality. For full reference, consult the PEtab reference. After finishing the implementation of the PEtab problem, its correctness can be verified using the PEtab library (see https://github.com/PEtab-

dev/PEtab/blob/master/doc/example/example_petablint.ipynb for instructions). The PEtab problem can then be used as input to the supporting toolboxes to estimate the unknown parameters or calculate parameter uncertainties. Links to tutorials for the different tools can be found at the PEtab Github page (https://github.com/PEtab-dev/PEtab#petab-support-in-systems-biology-tools).
